# Supplementary material for: Causal interplay between lactose intolerance and gut microbiota: a combined bidirectional Mendelian randomization and in vivo validation study
Source: Front Nutr. 2026 Jun 1;13:1803337. doi: 10.3389/fnut.2026.1803337 (PMC13265576; doi:10.3389/fnut.2026.1803337)
Supplement: Supplementary file 1 [file Data_Sheet_1.zip › supplementary materials/Forward/forest plot/ebi-a-GCST90027787.finngen_R12_E4_LACTONAS.pdf]

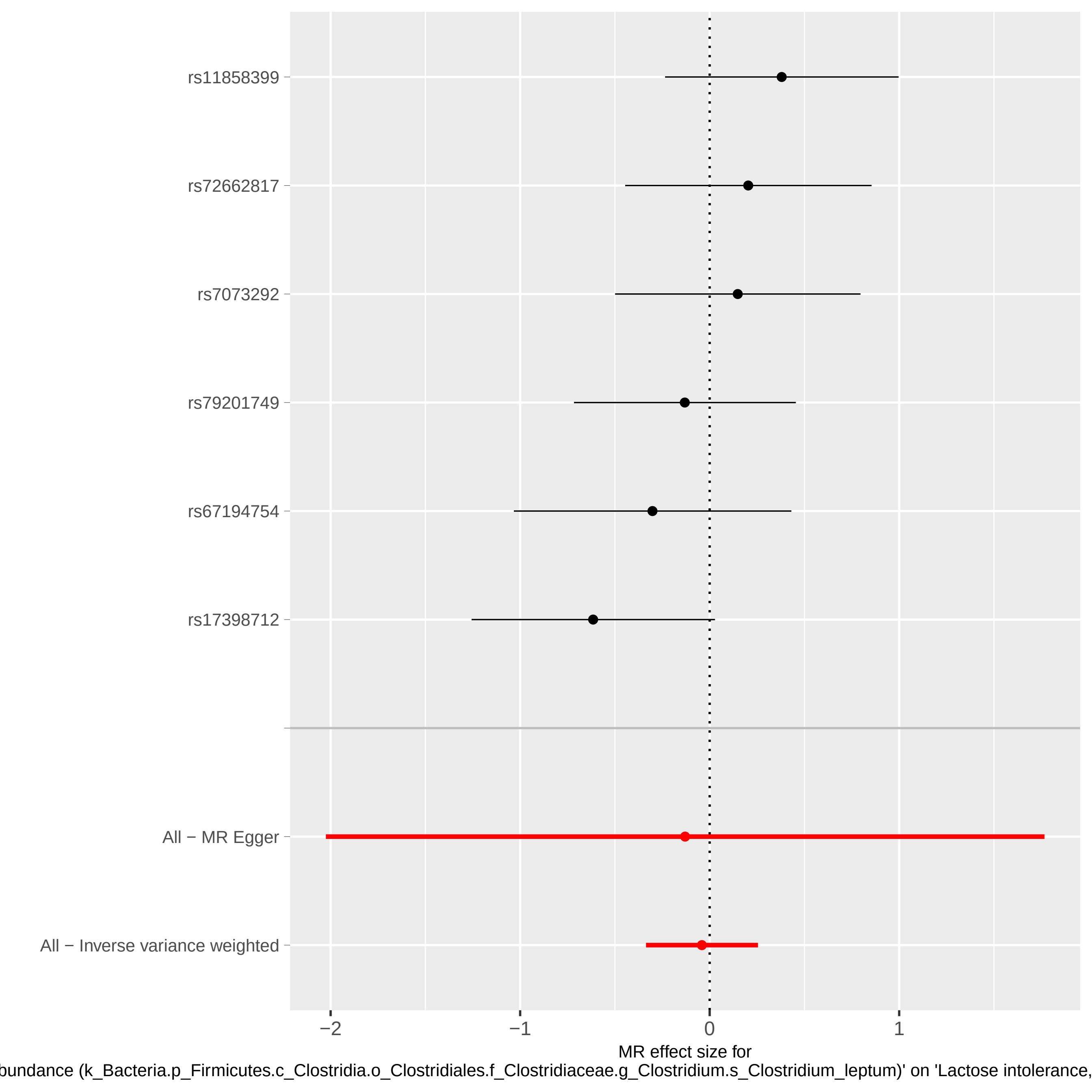

rs11858399
rs72662817
rs7073292
rs79201749
rs67194754
rs17398712
All − MR Egger
All − Inverse variance weighted
−2
−1
0
1
MR effect size for
bundance (k_Bacteria.p_Firmicutes.c_Clostridia.o_Clostridiales.f_Clostridiaceae.g_Clostridium.s_Clostridium_leptum)' on 'Lactose intolerance,
